# Intraocular Pressure-Lowering Effect of Latanoprost Is Hampered by Defective Cervical Lymphatic Drainage

**DOI:** 10.1371/journal.pone.0169683

**Published:** 2017-01-12

**Authors:** Young Kook Kim, Kyeong Ik Na, Jin Wook Jeoung, Ki Ho Park

**Affiliations:** 1 Department of Ophthalmology, Seoul National University Hospital, Seoul, Korea; 2 Department of Ophthalmology, Seoul National University College of Medicine, Seoul, Korea; Oregon Health and Science University, UNITED STATES

## Abstract

**Purpose:**

To evaluate whether defects in cervical lymphatic drainage influence the intraocular pressure (IOP)-lowering effect of latanoprost in patients with primary open-angle glaucoma (POAG) who have undergone unilateral radical neck dissection (uRND).

**Methods:**

We enrolled (1) bilateral POAG patients who had started (bilateral) latanoprost (0.005%) monotherapy prior to their uRND and (2) treatment-naïve, bilateral glaucoma suspects (GSs) who had undergone the same surgery. We compared the eyes ipsilateral to the uRND with their fellow eyes in terms of the changes in IOP between the baseline (prior to the uRND) and the follow-up visits (1, 3, and 6 months after the uRND).

**Results:**

The study involved 22 eyes of 11 POAG patients and 14 eyes of 7 GSs. In the POAG patients, IOP had increased significantly after surgery in the eyes ipsilateral to the uRND (from 14.7±1.4mmHg to 17.1±2.2mmHg; P = 0.007). Interestingly, in the eyes contralateral to the uRND, IOP had not changed significantly after surgery (from 14.2±1.8mmHg to 14.4±2.0mmHg; P = 0.826). In GSs, the eyes ipsilateral to the uRND did not differ significantly from their fellow eyes in terms of post-operative IOP change (ipsilateral value: 0.3±0.5mmHg, fellow eyes: -0.1±0.7mmHg; P = 0.242).

**Conclusion:**

In the POAG patients, IOP had increased significantly in the eyes ipsilateral to the uRND. However, it had not changed significantly in the eyes contralateral to the surgery or in the eyes of the GSs. These findings suggest that, latanoprost works, at least in part, by enhancing outflow from the aqueous humor via the uveolymphatic pathway.

## Introduction

Glaucoma is a chronic progressive disease characterized by optic nerve damage and visual field loss. To preserve sight in glaucoma patients, it is crucial to lower intraocular pressure (IOP).[1, 2] For this reason, investigators have mainly focused on improving fluid drainage from the eye using both pharmaceutical and surgical methods. Typically, aqueous humor is thought to drain from the eye via the trabecular meshwork and/or uveoscleral outflow pathways.[3] Recently, however, lymphatic vessels have been discovered in the human uvea itself, and animal studies have shown lymphatic fluid outflow through these vessels.[4–6] Additionally, there has been a report to the effect that regional lymphatic stasis and the absence of collateral drainage to the contralateral side significantly reduce the aqueous humour outflow and lead to bilateral, secondary IOP elevation.[7]

The prostaglandin (PG) F2a analogue such as latanoprost is one of the most widely prescribed topical eye medications for glaucoma. It lowers IOP by (1) stimulating the PG F2a receptors in the ciliary muscle, thereby increasing outflow,[8, 9] (2) widening connective tissue spaces,[10, 11] and (3) increasing uveoscleral outflow.[12, 13] However, PG F2a analogues are known to contract lymphatic vessels as well.[14, 15] In a study, the lymphatic drainage rate from the anterior chamber into the submandibular lymph node was found to be greater in latanoprost-treated mice than in controls.[16] Hence, it is thought that latanoprost might work, in part, by acting on the lymphatic channels in the eye.

Regardless of the mechanism, topical latanoprost effectively controls IOP.[17, 18] However, a subgroup of patients with glaucoma are unresponsive or poorly-responsive to latanoprost monotherapy. In one study, 25% of patients using latanoprost did not exhibit a “clinically meaningful” reduction in IOP.[19] We have hypothesized that the response to latanoprost monotherapy is affected by the drainage function of the lymphatic system. In the present study, therefore, we retrospectively investigated IOP in (1) latanoprost-treated patients with bilateral primary open-angle glaucoma (POAG) and (2) non-treated, bilateral glaucoma suspects (GSs), all of whom had subsequently undergone unilateral radical neck dissection (uRND), in order to compare IOP in the eyes ipsilateral to the uRND with their fellow eyes. The primary objective of this study was to evaluate the change in mean IOP between the baseline (when the cervical lymphatic drainage system was intact) and follow-up (when the system was defective).

## Methods

The study was approved by the Institutional Review Board of Seoul National University Hospital and followed the tenets of the Declaration of Helsinki. Due to the retrospective analysis of data which had already been obtained in routine clinical taking care of patients, the ethics committee decided that an informed consent signed by the individual patients was not necessary. Patient records/information was anonymized and de-identified prior to analysis.

### Study subjects

We enrolled (1) patients with bilateral POAG who had received pharmaceutical treatment for ocular hypotension (bilateral latanoprost 0.005% monotherapy) and (2) patients with bilateral GS who had never been treated with IOP-lowering medication. Using the customized data-sorting system for medical records (Clinical Data Warehouse; Bestcare software of SNUH, Seoul, South Korea), we consecutively selected those patients who had undergone uRND at the Head and Neck Cancer Clinic between January 2006 and February 2015.

All of the patients had undergone a complete ophthalmic examination including best-corrected visual acuity (logMAR) assessment, refraction, slit-lamp biomicroscopy, gonioscopy, dilated stereoscopic examination of the optic disc, red-free retinal nerve fiber layer (RNFL) photography, optic nerve head imaging by Cirrus DH-OCT (Carl Zeiss Meditec), and a central 30–2 threshold test of the Humphrey Visual Field (VF; HFA II; Humphrey Instruments Inc., Dublin, CA, USA).

An eye with GS was defined as that with one of the following findings: (1) a VF abnormality consistent with glaucoma, (2) an optic nerve or RNFL suggestive of glaucoma (enlarged or asymmetric cup/disc ratio, notching or narrowing of the neuroretinal rim, disc hemorrhage, or suspicious alteration in the RNFL), (3) an IOP higher than 21 mmHg. If two or more of these findings were present, the eye was diagnosed with POAG. Glaucomatous VF defects were defined as (1) a cluster of three points with probabilities less than 5% in at least one hemifield on the pattern deviation map, including at least one point with a probability less than 1%, (2) a cluster of two points with a probability less than 1% in at least one hemifield on the pattern deviation map, (3) glaucomatous Hemifield Test results outside the normal limits, or (4) a pattern standard deviation beyond 95% of the normal limits, as confirmed by at least two reliable examinations (false-positives/negatives: < 15%, fixation losses: < 15%).

Individuals were excluded on the basis of the following criteria: (1) secondary cause of glaucomatous optic neuropathy, (2) history of intraocular surgery (except cataract surgery) or ocular laser treatment (except Nd:YAG posterior capsulotomy), and (3) any neurological or systemic diseases that can affect retinal and VF results.

### Latanoprost monotherapy

All of the POAG patients had started latanoprost 0.005% monotherapy bilaterally more than 3 months before undergoing uRND, and had continued it for more than 6 months after the uRND. For patients who had been administered other glaucoma drugs, latanoprost monotherapy was started after a withdrawal period of at least 1 month. Patients who had presented with adverse events after the start of latanoprost monotherapy, or who had started any treatments other than latanoprost (such as anti-glaucoma eye drops, laser therapy, or glaucoma surgery), were eliminated from the study as dropouts. Also eliminated as dropouts were GSs who had ever been treated with IOP-lowering medication.

### uRND

Unilateral radical neck dissection (uRND) involves the unilateral removal of cervical lymph nodes from levels I through V, together with the spinal accessory nerve, internal jugular vein, and sternocleidomastoid muscle. The aim of this procedure is to remove lymph nodes into which cancer cells might have migrated. Thus, the procedure is carried out in cases of clinically or radiologically diagnosed lymph-node metastasis or as part of curative surgery in cases where the risk of occult nodal metastasis is deemed sufficiently high. In the present study, all of the patients treated using the uRND had also undergone surgery for a primary tumor (carcinoma of the mouth, oropharynx, hypopharynx, larynx, or thyroid). In some cases, one or more of the non-lymphatic structures (spinal accessory nerve, internal jugular vein and sternocleidomastoid muscle) were preserved, which procedures are defined as modified uRNDs.

### Pre- and post-operative follow-up IOP

For the POAG patients who had been treated using latanoprost monotherapy, the pre-operative mean IOP was defined as the mean of the IOP values obtained 1 month after the start of medical therapy and at each visit prior to the uRND; for the GSs, it was defined as the mean of the IOP values obtained at the initial visit and at each visit prior to the uRND. For all of the patients, IOP was measured 1, 3, and 6 months after surgery. All of these measurements were performed in the morning (from 9:00 am to 11:30 am) using Goldman applanation tonometry with the patient in a sitting position at the slit lamp. All of the study data were reviewed and analyzed by an author (Y.K.K) masked to all other information including the diagnosis of glaucoma and the side of the neck dissection.

### Statistical analysis

The nominal variables was analyzed using the Fisher exact test. ANOVA and the students t-test were employed for overall and pairwise comparisons of the continuous variables. Comparisons of overall mean IOP changes between fellow eyes were conducted using 1-way ANOVA for repeated measures. Because there were significant inter-group differences in baseline IOP, ANCOVA was utilized to test for mean IOP differences among the groups from the baseline. Statistical analysis was performed using the SPSS statistical package (SPSS 18.0; Chicago, IL, USA). A *P* value less than 0.05 was considered statistically significant.

## Results

### Study subjects

We initially distinguished 28 eyes of 14 patients with POAG and 20 eyes of 10 treatment-naive GSs, all of whom had undergone uRND. On the basis of a medical record review, we then recruited, for the present study, 22 POAG eyes of 11 patients, and 14 eyes of 7 GSs, which fulfilled the eligibility criteria. The POAG patients’ mean age was 54.72 ± 6.08 (38–71) years; seven were men (63.6%) and four were women (36.4%). The GSs’ mean age was 57.29 ± 5.50 (35–68) years; five were men (71.4%) and two were women (28.6%). The eyes in each group were differentiated according to their laterality to the uRND; that is, eyes were either ipsilateral or contralateral to the uRND.

In the POAG group, there was no significant difference in laterality (P = 0.394); furthermore, the ipsilateral and contralateral eyes did not differ in terms of visual acuity (P = 0.853), VF mean deviation (P = 0.683), refractive error (P = 0.897), central corneal thickness (P = 0.950), circumpapillary RNFL thickness (P = 0.430), baseline pre-treated IOP (P = 0.700) or baseline treated IOP (P = 0.501). Latanoprost monotherapy reduced the mean IOP by 3.7 ± 1.9 mmHg (from 18.2 ± 2.4 mmHg to 14.5 ± 1.7 mmHg); this effect did not differ significantly between fellow eyes (P = 0.812).

In the GS group, there was no significant difference in the laterality (P = 1.000), and the ipsilateral and contralateral eyes did not differ in terms of visual acuity (P = 0.797), VF mean deviation (P = 0.829), refractive error (P = 0.766), central corneal thickness (P = 0.820), circumpapillary RNFL thickness (P = 0.424) or baseline IOP (P = 0.828). The demographic and clinical characteristics of the POAG group are presented in Table 1, while those of the GS group are provided in Table 2.

**Table 1:** Baseline characteristics of POAG eyes ipsilateral to the uRND and in POAG eyes contralateral to the uRND.

Characteristics	Ipsilateral to the uRND (n = 11)		Contralateral to the uRND (n = 11)	*P*
Age		54.72 ± 6.08		-
Gender (Male / Female)		7 / 4		-
Laterality (OD / OS)		7 / 4	4 / 7	0.394
Visual acuity (logMAR)		0.19 ± 0.42	0.23 ± 0.57	0.853
Visual field mean deviation (dB)		-8.61 ± 3.23	-9.17 ± 3.10	0.683
Refractive error (spherical equivalent; diopters)		-0.34 ± 2.59	-0.52 ± 3.76	0.897
Central corneal thickness (μm)		525.1 ± 30.4	524.2 ± 29.9	0.950
Circumpapillary RNFL thickness (μm)		68.3 ± 11.5	64.2 ± 12.0	0.430
Pretreat IOP		18.3 ± 2.5	17.9 ± 2.3	0.700
Treat IOP		14.7 ± 1.4	14.2 ± 1.8	0.501

POAG, primary open-angle glaucoma; uRND, unilateral radical neck dissection; logMAR, logarithm of the Minimum Angle of Resolution; dB, decibel; RNFL, retinal nerve fiber layer; IOP, intraocular pressure

**Table 2:** Baseline characteristics of GS eyes ipsilateral to the uRND and in GS eyes contralateral to the uRND.

Characteristics	Ipsilateral to the uRND (n = 7)	Contralateral to the uRND (n = 7)	*P*
Age	57.29 ± 5.50		-
Gender (Male / Female)	5 / 2		-
Laterality (OD / OS)	3 / 4	4 / 3	1.000
Visual acuity (logMAR)	0.15 ± 0.33	0.20 ± 0.38	0.797
Visual field mean deviation (dB)	-0.32 ± 1.12	-0.19 ± 1.08	0.829
Refractive error (spherical equivalent; diopters)	-0.78 ± 2.13	-0.36 ± 2.97	0.766
Central corneal thickness (μm)	535.3 ± 28.7	531.1 ± 38.3	0.820
Circumpapillary RNFL thickness (μm)	89.5 ± 7.8	86.4 ± 6.1	0.424
IOP (mmHg)	17.4 ± 2.3	17.0 ± 2.8	0.828

GS, glaucoma suspect; uRND, unilateral radical neck dissection; logMAR, logarithm of the Minimum Angle of Resolution; dB, decibel; RNFL, retinal nerve fiber layer; IOP, intraocular pressure

### Post-operative IOP changes in POAG eyes

**Fig 1:**
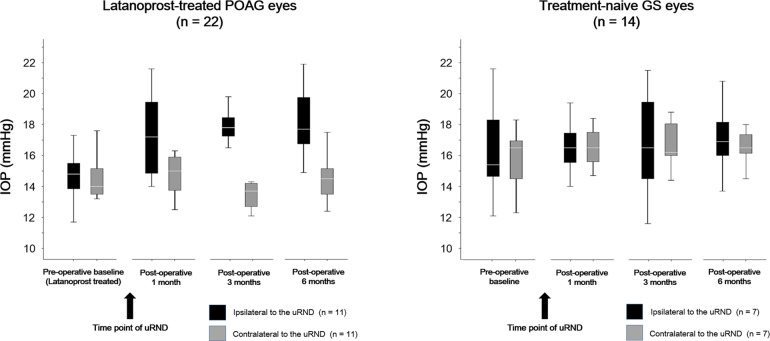
Comparisons of eyes ipsilateral to unilateral radical neck dissection (uRND) with fellow eyes in terms of changes in intraocular pressure (IOP) between pre-operative baseline (prior to uRND) and post-operative follow-up visits (1, 3, and 6 months after uRND). POAG = primary open-angle glaucoma, GS = glaucoma suspect. ^*^ *P* = 0.015, ^**^ *P* = 0.007, ^***^ *P* = 0.004 (when comparing post-operative IOP to pre-operative baseline).

Among the treated POAG patients, the mean IOP significantly increased immediately after surgery (from 14.5 ± 1.7 mmHg to 16.2 ± 1.8 mmHg; P = 0.034). Interestingly, the POAG eyes ipsilateral to the uRND showed a greater IOP increase than did their fellow eyes (2.4 ± 0.8 mmHg *vs*. 0.2 ± 0.7 mmHg; P < 0.001; Table 3). Specifically, in the POAG eyes ipsilateral to the uRND, the mean IOP values 1, 3, and 6 months after surgery were 115.0% (P = 0.015), 113.6% (P = 0.007), and 120.4% (P = 0.004) of the pre-operative value, respectively (avg.: 116.3% [P = 0.007]). Among the POAG eyes contralateral to the uRND, those values were 105.6% (P = 0.317), 96.5% (P = 0.566), and 101.4% (P = 0.833) of the pre-operative value, respectively (avg.: 101.4% [P = 0.826]). The mean IOP was more likely to be increased in the POAG eyes ipsilateral to the uRND than in their fellow eyes; specifically, IOP had increased by 15% or more in 81.8% (9/11) of the ipsilateral eyes and 9.1% (1/11) of the contralateral eyes (P < 0.001; Fig 1). Table 4 shows the relevant data on each of the POAG patients (gender, age, systemic comorbidities, IOP, site of primary tumor, side of uRND, and type of adjuvant therapies).

**Table 3:** IOP changes in latanoprost-treated-POAG eyes after the uRND.

	POAG eyes (*n = 22*)						
	Ipsilateral to the uRND (*n = 11*)			Contralateral to the uRND (*n = 11*)			*P*
Characteristics	IOP (*mmHg*)	Change from pre-operative baseline		IOP (*mmHg*)	Change from pre-operative baseline		
		*%*	*P*		*%*	*P*	
Pre-operative baseline	14.7 ± 1.4	-	-	14.2 ± 1.8	-	-	0.541
Post-operative period	17.1 ± 2.2	116.3	0.007	14.4 ± 2.0	101.4	0.826	0.007
• *1 month*	16.9 ± 2.3	115.0	0.015	15.0 ± 1.9	105.6	0.317	0.047
• *3 months*	16.7 ± 1.6	113.6	0.007	13.7 ± 1.8	96.5	0.566	<0.001
• *6 months*	17.7 ± 2.7	120.4	0.004	14.4 ± 2.2	101.4	0.833	0.005

IOP, intraocular pressure; POAG, primary open-angle glaucoma; uRND, unilateral radical neck dissection

**Table 4:** Patient summary.

Patient^*^	Gender / age,^*^ (y)	Comorbidities		Glaucoma				Primary tumor		uRND		Adjuvant therapy		Post-uRND IOP^***^	
		DM	HTN	Diagnosis		Mean F/U IOP^**^		Location	Diagnosis	Laterality	Extent	Chemotherapy	Radiation therapy	OD	OS
				OD	OS	OD	OS								
1	M / 56	+	-	POAG	POAG	16.6	16.9	Thyroid	Papillary carcinoma	OD	RND	-	-	20.4	17.0
2	M / 58	+	+	POAG	POAG	14.1	12.6	Oropharynx	Squamous cell carcinoma	OD	RND	+	+	16.1	12.6
3	M / 66	-	-	POAG	POAG	14.3	12.7	Thyroid	Papillary carcinoma	OD	RND	+	-	15.4	13.7
4	M / 44	-	-	POAG	POAG	15.7	15.3	Tongue	Squamous cell carcinoma	OD	RND	+	-	18.9	16.3
5	F / 47	-	-	POAG	POAG	17.5	17.5	Thyroid	Papillary carcinoma	OD	Modified RND	+	-	19.9	17.8
6	F / 50	-	-	POAG	POAG	13.6	14.6	Thyroid	Papillary carcinoma	OD	RND	+	-	15.2	15.0
7	M / 59	+	+	POAG	POAG	13.2	12.2	Thyroid	Papillary carcinoma	OD	Modified RND	+	-	15.0	12.0
8	F / 56	+	-	POAG	POAG	14.4	14.9	Thyroid	Squamous cell carcinoma	OS	Modified RND	+	-	13.8	17.3
9	F / 55	-	-	POAG	POAG	14.5	14.5	Thyroid	Papillary carcinoma	OS	RND	-	-	14.1	16.9
10	M / 58	+	+	POAG	POAG	12.1	12.9	Thyroid	Papillary carcinoma	OS	Modified RND	+	-	12.1	13.8
11	M / 53	-	-	POAG	POAG	14.0	14.8	Thyroid	Papillary carcinoma	OS	Modified RND	+	-	13.6	19.2
12	M / 59	-	-	GS	GS	16.3	15.9	Thyroid	Papillary carcinoma	OD	Modified RND	-	-	15.9	15.7
13	M / 63	+	-	GS	GS	14.9	14.1	Oropharynx	Squamous cell carcinoma	OD	RND	+	-	14.6	14.1
14	F / 65	+	+	GS	GS	19.6	19.2	Thyroid	Squamous cell carcinoma	OD	Modified RND	+	-	20.2	19.0
15	F / 49	-	-	GS	GS	18.4	18.2	Thyroid	Papillary carcinoma	OS	RND	-	-	18.7	18.6
16	M / 55	-	-	GS	GS	18.7	18.8	Thyroid	Papillary carcinoma	OS	Modified RND	+	-	18.5	19.2
17	M / 54	-	-	GS	GS	13.0	14.1	Thyroid	Squamous cell carcinoma	OS	Modified RND	+	-	13.1	15.0
18	M / 56	-	+	GS	GS	19.4	19.7	Thyroid	Papillary carcinoma	OS	Modified RND	+	-	19.3	20.3

* Age at operation.

** Mean treat-IOP for glaucoma patients and mean untreat-IOP for glaucoma suspects.

*** Mean IOP during the post-op 6 months.

DM, diabetes mellitus; HTN, hypertension; OD, oculus dexter; OS, oculus sinister; uRND, unilateral radical neck dissection; LN, lymph node; POAG, primary open-angle glaucoma; GS, glaucoma suspect.

### Post-operative IOP changes in GS eyes

Among the GSs, the mean IOP did not change significantly between the pre-operative baseline and the post-operative period (17.2 ± 3.4 mmHg *vs*. 17.3± 3.2 mmHg; P = 0.957). The mean IOP value in the GS eyes ipsilateral to the uRND increased slightly after surgery (from 17.4 ± 3.1 mmHg to 17.7 ± 3.0 mmHg; P = 0.857). However, in the contralateral eyes, the IOP decreased slightly after surgery (from 17.0 ± 3.6 mmHg to 16.9 ± 3.4 mmHg; P = 0.649; Table 5 and Fig 1). Specifically, in the GS eyes ipsilateral to the uRND, the mean IOP values 1, 3, and 6 months after surgery were 100.6% (P = 0.956), 103.4% (P = 0.398), and 102.3% (P = 0.782), respectively. In the GS eyes contralateral to the uRND meanwhile, those IOP values were 101.2% (P = 0.850), 100.0% (P = 0.962), and 98.8% (P = 0.849), respectively. Table 4 shows the relevant data on each of the GSs (gender, age, systemic comorbidities, IOP, site of primary tumor, side of uRND, and type of adjuvant therapies).

**Table 5:** IOP changes in non-treated-GS eyes after uRND.

	GS eyes (*n = 14*)						
	Ipsilateral to uRND (*n = 7*)			Contralateral to uRND (*n = 7*)			*P*
Characteristics	IOP (*mmHg*)	Change from pre-operative baseline		IOP (*mmHg*)	Change from pre-operative baseline		
		*%*	*P*		*%*	*P*	
Pre-operative baseline	17.4 ± 2.3	-	-	17.0 ± 2.8	-	-	0.828
Post-operative period	17.7 ± 2.5	101.7	0.857	16.9 ± 2.6	99.4	0.958	0.649
• *1 month*	17.5 ± 2.7	100.6	0.956	17.2 ± 3.1	101.2	0.948	0.850
• *3 months*	18.0 ± 2.5	103.4	0.398	17.0 ± 2.5	100.0	0.962	0.469
• *6 months*	17.8 ± 2.3	102.3	0.782	16.7 ± 2.9	98.8	0.849	0.524

IOP, intraocular pressure; GS, glaucoma suspect; uRND, unilateral radical neck dissection

### Case study

A 63-year-old man was diagnosed with bilateral POAG (Humphrey VF mean deviation: -5.67 dB in right eye and -6.09 dB in left eye) in April 2012. His untreated IOP was 18 mmHg in the right eye and 17 mmHg in the left eye. He was prescribed 0.005% latanoprost in both eyes, and the mean treated IOP was 14.2 mmHg in the right eye and 14.4 mmHg in the left eye. In September 2013, he was diagnosed with papillary thyroid cancer on the right lobe. So, he underwent a total thyroidectomy combined with right-sided uRND. One month after surgery, the IOP in the right eye had increased from 14.2 mmHg to 16 mmHg; however, there was no significant change in the left eye (from 14.4 mmHg to 14.5 mmHg). Three (3) months after surgery, the IOP was 17.5 mmHg in the right eye and 14.5 mmHg in the left eye; 6 months after surgery, the values were 17 mmHg in the right eye and 14 mmHg in the left eye. Thus, for both eyes, the patient’s medication was changed from 0.005% latanoprost to 0.5% timolol. One month later, the IOP in the right eye had decreased from 17.5 mmHg to 15 mmHg; after 6 and 12 months, the IOP in that eye was 14 mmHg and 14.5 mmHg, respectively.

## Discussion

In the present study, the POAG patients were prescribed 0.005% latanoprost bilaterally and had continuously used it before and after uRND. In the eyes ipsilateral to the uRND, IOP was significantly elevated after surgery. Conversely, there were no significant IOP changes in the fellow eyes. To our knowledge, this is the first study to demonstrate the effect of defective cervical lymphatic drainage on IOP in treated glaucoma patients.

Lymphatic vessels are well-known to drain extracellular fluid[20–23]; however, until recently, they were believed to be absent from the eye.[24, 25] This belief raised several perplexing questions. For instance, how does the normal aqueous humor remain virtually protein free despite the high metabolic activity of the surrounding ocular tissues? And, how is interstitial fluid removed from ocular tissues?[4] Previous pioneering studies have posited the chronic cervical lymphostasis effect on the regions primarily drained by prelymphatics (including the retina, papilla, iris, choroid and Circle of Willis).[26, 27] Advances in lymphatic research with lymphatic-specific markers have enabled investigators to identify ciliary-body lymphatic circulation[5] that might allow aqueous humor to flow out of the anterior chamber.[4, 6] On this basis, the lymphatic network of the ciliary body was hypothesized, as the “uveolymphatic pathway,”[5, 28] to be the third route of aqueous humor drainage.

Lymphatic vessels respond to PG F2a analogues,[29–31] which are potent inducers of contractility in mesenteric lymphatic vessels.[14, 15] In one study, the lymphatic drainage rate from the anterior chamber into the submandibular lymph node was found to be greater in latanoprost-treated mice than in controls.[16] Therefore, lymphatic drainage can be thought to be an active process driven by peristalsis[32, 33]; it follows, moreover, that this process can be stimulated pharmacologically.[15, 33] Thus prompted, we investigated whether the IOP alters in cases of defective cervical lymphatic drainage. Specifically, because the lymphatic vessels can be stimulated by PG F2a analogues, we studied whether the IOP in latanoprost-treated patients is changed in cases of lymphatic channel deterioration or blockage.

Our most significant finding was that, in patients with POAG, IOP increased more after surgery in eyes ipsilateral to the uRND. Additionally, those eyes were more likely than their fellow eyes to have increased IOP after surgery. This constitutes circumstantial evidence that the IOP-lowering effect of latanoprost was influenced by defective cervical lymphatic drainage, such as that caused by uRND, and that latanoprost had worked, at least in part, by enhancing aqueous humor outflow via the uveolymphatic pathway.

To investigate the pure effect of defective cervical lymphatic drainage on IOP, we recruited treatment-naïve bilateral GSs who had undergone uRND. Interestingly, immediately after surgery, the mean IOP had not changed significantly in the GSs. Furthermore, there were no significant differences in post-operative-IOP change between GS eyes ipsilateral to the uRND and their fellow eyes. These findings could be explained in two ways. First, aqueous humor might be drained through the uveolymphatic pathway much less than through the trabecular meshwork or uveoscleral outflow pathways. Second, treatment-naïve patients might not be affected as much by defective lymphatic drainage, because the uveolymphatic pathway is an active process that might need glaucoma medication for its stimulation.

In the present study, 7 (63.6%) of 11 POAG patients underwent uRND on their right eyes and the remaining 3, on their left eyes. As the microanatomy of the right cervical lymphatic system is different from that of the left cervical lymphatic system,[34] we re-evaluated whether the IOP-lowering effect of latanoprost was different between the right and left eyes of our study subjects. In the results, there was no significant difference (right eye: 0.3±0.5mmHg, left eye: -0.1±0.7mmHg; P = 0.242).

This study has three clinical significances. First, the evidence of a lymphatic outflow system in human eyes has expanded our understanding of how fluid and particulate materials—such as proteins—move out of the eye, as well as how IOP might be regulated. Second, physicians in future might have to evaluate lymphatic drainage function as a component of IOP management. For example, in patients suffering from decreased lymphatic drainage into the cervical area, such as occurs in lymphatic diseases (i.e., lymphadenopathy, lymphedema, or lymphangitis), the IOP-lowering effects of latanoprost would be altered by these co-morbidities. Finally, the present findings collectively point to lymphatics as a novel target for glaucoma treatment. Clinicians can expand pharmacological IOP-lowering treatment to take into account this third outflow pathway by stimulate them pharmacologically.

### Study limitations

The findings of this study must be interpreted in the light of several points.

1. The sample size was small, and the study was performed retrospectively.
2. Most of our glaucoma patients had a baseline IOP of 21 mmHg or lower, as well as early-to-moderate glaucoma; therefore, our results might not be relevant to other populations, such as patients with a baseline IOP higher than 21 mmHg or those with advanced glaucoma.
3. Changes to the optic nerve head [35] or visual functions [36] upon blockage of the cervical lymphatic channel have been reported. However, in this study, there were no significant interval changes in the optic disc parameters or other glaucoma parameters (i.e., VF mean deviation or OCT RNFL thickness) after the uRND. This might have been due to the fact that we investigated relatively short-term changes only (for all of the patients, IOP was measured 1, 3, and 6 months after surgery). Although there were significant differences in post-operative IOP changes between fellow eyes, the pattern of IOP change might also differ more than 6 months after surgery.
4. The thyroid disorder potentially increases the risk of glaucoma [37] and can have an effect on the diurnal variation of IOP.[38] Also, the thyroid function and IOP could have responded to corticosteroid that was applied after surgery.[39] In fact, 9 (81.8%) of 11 POAG patients and 6 (85.7%) of 7 GS patients had the thyroid tumor in this study. Therefore, the pattern of IOP change could have been affected not only by uRND but also by post-operative thyroid function and the use of corticosteroid.
5. Our patients had undergone uRND that involved the removal of the spinal accessory nerve, sternocleidomastoid muscle, and internal jugular vein and the resection of primary tumor. The study subjects had also undergone adjuvant therapies such as chemotherapy or radiotherapy. These additional procedures could have affected the IOP during the post-operative period.

In conclusion, in latanoprost-treated POAG patients, IOP increased significantly after surgery in the eyes ipsilateral to the uRND; however, it remained similar in their fellow eyes as well as in the treatment-naïve GS eyes. These findings signify that in human eyes, latanoprost might work, at least in part, by enhancing aqueous humor outflow via the hypothesized uveolymphatic pathway. Studying the lymphatics in normal and glaucomatous eyes, as well as under pharmacological manipulation, could lead to the discovery of helpful new approaches to IOP management.
